# Development and Validation of a Rapid LC–MS/MS Method for Plasma Analysis of Ketamine, Norketamine, Dehydronorketamine, and Hydroxynorketamine

**DOI:** 10.1002/bmc.70171

**Published:** 2025-07-21

**Authors:** Jan Thomann, Selina Kraus, Livio Erne, Severin B. Vogt, Matthias E. Liechti, Dino Luethi

**Affiliations:** ^1^ Division of Clinical Pharmacology and Toxicology, Department of Biomedicine University Hospital Basel Basel Switzerland; ^2^ Division of Clinical Pharmacology and Toxicology, Department of Pharmaceutical Sciences University of Basel Basel Switzerland; ^3^ Division of Clinical Pharmacology and Toxicology, Department of Clinical Research University Hospital Basel Basel Switzerland

**Keywords:** bioanalysis, clinical study, liquid chromatography, mass spectrometry, pharmacokinetics

## Abstract

Ketamine, a well‐established dissociative anesthetic, has recently gained significant attention for its rapid‐acting antidepressant effects, particularly in treatment‐resistant depression. In this study, we developed and validated a state‐of‐the‐art liquid chromatography–tandem mass spectrometry (LC–MS/MS) method for the bioanalysis of ketamine and its metabolites, norketamine, dehydronorketamine (DHNK), and (2R,6R)‐hydroxynorketamine (HNK), in human plasma. The method features a small sample volume, a streamlined protein precipitation protocol, and a rapid sample runtime. The mobile phase gradient is composed of an aqueous ammonium hydrogen carbonate solution and pure acetonitrile. Using positive electrospray ionization, linear quantification ranges of 1–1,000 ng/mL were established for ketamine and norketamine, while ranges of 0.25–100 ng/mL for DHNK and 2.5–1,000 ng/mL for (2R,6R)‐HNK were achieved. The method demonstrated high accuracy, precision, selectivity, and sensitivity, along with consistent matrix effects, efficient extraction recovery, and analyte stability. Finally, the method was successfully applied to assess the pharmacokinetics of six clinical trial participants. Overall, this LC–MS/MS method offers a robust and efficient approach for the achiral quantification of ketamine and its metabolites in human plasma. Its minimal sample preparation and reduced analytical runtime make it particularly well‐suited for clinical studies, drug monitoring, and forensic investigations.

## 1 Introduction

The arylcyclohexylamine ketamine is a dissociative anesthetic that has gained increasing interest in both clinical and research contexts. Originally developed as a safer alternative to phencyclidine (PCP), ketamine was approved for human use by the US Food and Drug Administration (FDA) in 1970 and has since been widely utilized in both human and veterinary medicine, primarily for induction and maintenance of anesthesia (Dinis‐Oliveira 2017; Hirota and Lambert 2022). Ketamine contains a chiral center and is typically found as a racemic mixture. However, the isomer S(+)‐ketamine is often used as an anesthetic due to its greater analgesic potency and less drowsiness, lethargy, and cognitive impairment (Dinis‐Oliveira 2017; Hirota and Lambert 2022; Passie et al. 2021).

Pharmacologically, ketamine acts primarily as a noncompetitive *N*‐methyl‐D‐aspartate (NMDA) receptor antagonist. It has been demonstrated to bind to the allosteric PCP binding site within the channel pore. The mechanism of action of ketamine involves the inhibition of NMDA receptor activity by reducing calcium ion influx. This results in the disruption of the transmission of pain signals and the exertion of dissociative, anesthetic, amnesic, analgesic, and antidepressant effects (Dinis‐Oliveira 2017; Schep et al. 2023; Zanos and Gould 2018; Zanos et al. 2018). S(+)‐ketamine has been shown to exhibit approximately fourfold higher affinity for the PCP binding site than R(−)‐ketamine, thereby explaining its greater potency in producing these effects (Zanos et al. 2018). In addition to its NMDA antagonism, ketamine has been shown to interact with opioid receptors, monoaminergic pathways, cholinergic receptors (both muscarinic and nicotinic acetylcholine receptors), and other targets, which contribute to its complex pharmacodynamic profile. In contrast to other anesthetics, ketamine does not relevantly interfere with 𝛾‐aminobutyric acid (GABA) receptors (Dinis‐Oliveira 2017; Hirota and Lambert 2022; Zanos and Gould 2018; Zanos et al. 2018).

Ketamine is stereoselectively metabolized primarily in the liver by cytochrome P450 (CYP) enzymes. The initial metabolic step is N‐demethylation to norketamine, primarily via CYP3A4 and CYP2B6, with contributions from CYP2C9, CYP2C19, CYP2A6, and CYP2D6 (Dinis‐Oliveira 2017; Kamp, Jonkman, et al. 2020; Zanos et al. 2018). Norketamine, an active metabolite that also inhibits NMDA receptors, exhibits psychoactive and anesthetic properties. Norketamine is further metabolized by hydroxylation, mainly via CYP2B6 and CYP2A6, to form hydroxynorketamine (HNK), including isomers with antidepressant and analgesic effects such as (2R,6R)‐HNK and (2S,6S)‐HNK (Dinis‐Oliveira 2017; Kamp, Jonkman, et al. 2020; Zanos et al. 2018). Furthermore, the formation of dehydronorketamine (DHNK) can be observed either by nonenzymatic dehydration of the 5‐HNK isoform or directly via CYP2B6 from norketamine. HNK can also be O‐glucuronidated by uridine‐5′‐diphospho‐glucuronosyltransferases (UGTs) to form HNK‐glucuronides (Dinis‐Oliveira 2017; Kamp, Jonkman, et al. 2020; Sandbaumhüter and Thormann 2018; Zanos et al. 2018). Other minor metabolic pathways that have been reported include the hydroxylation of ketamine to hydroxyketamine and the formation of urinary phenolic metabolites of ketamine and norketamine (Dinis‐Oliveira 2017; Zanos et al. 2018).

Lately, ketamine has received renewed attention for its rapid‐acting antidepressant properties, particularly in treatment‐resistant major depressive disorder (MDD) and suicidal ideation (Glue et al. 2024; Hirota and Lambert 2022; Williams et al. 2019; Zolghadriha et al. 2024). At subanesthetic doses, typically administered intravenously or intranasally, ketamine has demonstrated significant and rapid mood improvement in clinical trials, often within hours of administration. This is in clear contrast to conventional antidepressants, which usually take weeks to achieve efficacy (Rosenblat et al. 2023; Williams et al. 2019; Zolghadriha et al. 2024). Furthermore, it has been suggested that the metabolites (2S,6S)‐HNK and (2R,6R)‐HNK exert an antidepressant effect but lack the typical side effects associated with ketamine (Loan Nguyen et al. 2024; Zanos et al. 2016; Zanos et al. 2018). These findings have sparked interest in the development of ketamine analogs and derivatives, including esketamine, which was FDA‐approved in 2019 as a nasal spray for depression in combination therapy and as a stand‐alone therapy in 2025 (Reuters 2025).

In recent years, a considerable number of LC–MS methods have been developed for the detection and quantification of ketamine and metabolites in various biological matrices, including plasma, serum, urine, feces, milk, cerebrospinal fluid, and brain tissue. Advanced methods also include enantiomeric separation using column switching and chiral columns (Harun et al. 2010; Hasan et al. 2017; Kurzweil et al. 2020; Moaddel et al. 2010; Rhee et al. 2021; Rochani et al. 2020; Rodriguez Rosas et al. 2003; Shahane et al. 2023; Toki et al. 2023). Sample preparation often involves laborious liquid–liquid or solid‐phase extraction. Moreover, the methods entail extensive sample runtimes, which are impractical for the analysis of a large number of study samples (Harun et al. 2010; Hasan et al. 2017; Kurzweil et al. 2020; Moaddel et al. 2010; Rhee et al. 2021; Rochani et al. 2020; Rodriguez Rosas et al. 2003; Shahane et al. 2023; Toki et al. 2023).

Consequently, the method developed and validated in this study was designed to optimize sample preparation by reducing labor‐intensive steps and decreasing the runtime of a single sample. This enhances its suitability for analyzing large numbers of clinical study samples. Furthermore, the incorporation of metabolites norketamine, DHNK, and (2R,6R)‐HNK provides comprehensive insights into ketamine's in vivo metabolism and pharmacokinetics. Finally, a subset of clinical study samples was analyzed to demonstrate method functionality.

## 2 Experimental

### 2.1 Chemicals, Reagents, and Reference Compounds

Ketamine hydrochloride (99.9% purity, 1 mg/mL free base in methanol), norketamine hydrochloride (99.5% purity, 1 mg/mL free base in methanol), DHNK hydrochloride (99.6% purity, 0.1 mg/mL free base in acetonitrile), and the deuteriated internal standards ketamine‐d_4_ hydrochloride (99.7% purity, 1 mg/mL free base in methanol) and norketamine‐d_4_ hydrochloride (99.8% purity, 0.1 mg/mL free base in methanol) were acquired from Cerilliant (Round Rock, TX, USA). (2R,6R)‐HNK (99.2% purity) and (2S,6S)‐HNK (99.1% purity) were obtained from Sigma‐Aldrich (St. Louis, MO, USA). LC–MS grade water, methanol, isopropanol, ammonium hydrogen carbonate, and ammonia solution 25% were provided by Merck (Darmstadt, Germany). Dimethyl sulfoxide (DMSO) and acetonitrile were obtained from Sigma‐Aldrich and Thermo Fisher Scientific (Waltham, MA, USA), respectively.

Blank human plasma was drawn from voluntary donors at the University Hospital Basel and collected into lithium heparin‐coated S‐Monovette tubes (Sarstedt, Nümbrecht, Germany) to prevent blood clotting. To yield the plasma from whole blood, the tubes were centrifuged at 1811 × *g* for 10 min (5810 R centrifuge, Eppendorf, Hamburg, Germany).

### 2.2 Stock Solution Preparation

For each analyte, two separate stock solutions were prepared: one for calibrators (CAL) and one for quality control (QC) samples. These samples were either directly obtained or prepared. Using the CAL or QC stock solutions, each containing 1 mg/mL ketamine, 1 mg/mL norketamine, 1 mg/mL (2R,6R)‐HNK, or 0.1 mg/mL DHNK, two stock mixtures were prepared in DMSO. These stock mixtures contained 200 μg/mL of ketamine, norketamine, and (2R,6R)‐HNK, and 20 μg/mL of DHNK. All stock solutions were stored at −20 °C.

### 2.3 Calibration and QC Sample Preparation

The 200‐μg/mL stock mixtures were serially diluted in DMSO. The dilution steps were performed to obtain samples with final concentrations ranging from 0.1 to 100 μg/mL for ketamine and norketamine, from 0.025 to 10 μg/mL for DHNK, and from 0.25 to 100 μg/mL for (2R,6R)‐HNK. Subsequently, the CAL and QC dilutions were used to spike pooled plasma from seven donors at a 1:100 (v/v) ratio. This resulted in CAL lines ranging from 1 to 1,000 ng/mL for ketamine and norketamine, from 0.25 to 100 ng/mL for DHNK, and from 2.5 to 1,000 ng/mL for (2R,6R)‐HNK. QC samples were prepared analogously to CAL samples. For each substance, four QC levels were included: the lower limit of quantification (LLOQ), low QC (LQC), mid QC (MQC), and high QC (HQC). The QC levels for ketamine and norketamine were set at 1, 2.5, 50, and 500 ng/mL; for DHNK at 0.25, 0.5, 5, and 50 ng/mL; for (2R,6R)‐HNK at 2.5, 5, 50, and 500 ng/mL.

### 2.4 LC–MS/MS Instrumentation and Settings

The analysis was performed using a high‐performance liquid chromatograph (HPLC, Shimadzu, Kyoto, Japan) coupled to an API 5500 triple quadrupole tandem mass spectrometer (MS, AB Sciex, Ontario, Canada). The HPLC system consisted of an autosampler (set to 10 °C), three pumps (A, B, and C), a system controller, two degassing units, and a column oven (set to 35 °C). Best chromatographic results were achieved using a Symmetry C18 column (3.5 μm, 100 Å, 75 × 4.6 mm, Waters, Milford, USA). Mobile phase A comprised a 20‐mM ammonium hydrogen carbonate solution in water, adjusted to pH 9 with 25% ammonia solution. Mobile phase B consisted of pure acetonitrile. Before and after each sample injection (10 μL injection volume), the autosampler port was purged with 500 μL washing solution (water, acetonitrile, methanol, and isopropanol 1:1:1:1, v/v).

**FIGURE 1:**
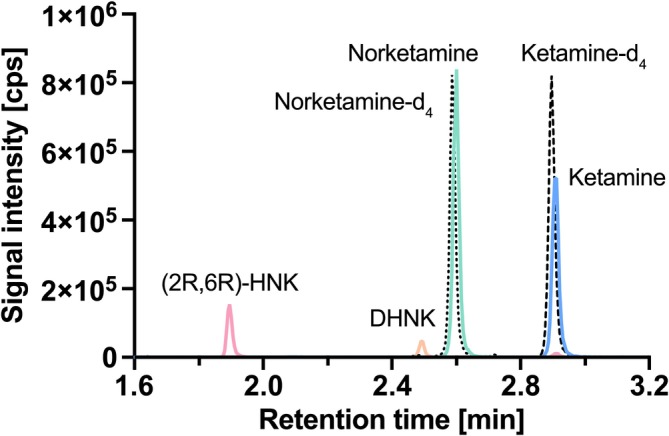
Chromatographic separation of ketamine (100 ng/mL), norketamine (100 ng/mL), dehydronorketamine (DHNK; 10 ng/mL), (2R,6R)‐hydroxynorketamine (HNK; 100 ng/mL), ketamine‐d_4_ (10 ng/mL), and norketamine‐d_4_ (20 ng/mL) in pooled human plasma.

The injected sample was first transported to a T‐union (located in front of the column) by pumps A and B, where it was diluted with mobile phase A by pump C (0.7 mL/min, 0–0.5 min). The initial total flow of pump A (90%) and pump B (10%) was set to 0.7 mL/min and was increased to 1.4 mL/min after 0.5 min. The concentration of mobile phase B was linearly raised from 10% at 0.5 min to 95% after 3.0 min. Subsequently, mobile phase B concentration remained at 95% for 1.0 min to wash the column. Next, the concentration of mobile phase B was returned to 10% until 4.5 min to recondition the column. The eluent was directed into the mass spectrometer from 1.4 to 3.5 min and otherwise directed into the waste. Using this method, ketamine had a retention time of 2.92 min, norketamine 2.61 min, DHNK 2.51 min, and (2R,6R)‐HNK 1.91 min. The chromatographic separation of all substances is shown in Figure 1.

The analytes were ionized using positive electron spray ionization (ESI) and detected with multiple reaction monitoring (MRM) in positive ion mode (Table 1). The mass spectrometer parameters were set as follows: source temperature (TEM), 500 °C; ion spray voltage (IS), 5500 V; nebulizer gas (ion source gas 1, GS1), 30 psi; heater gas (ion source gas 2, GS2), 60 psi; curtain gas (CUR), 30 psi; and collision gas (CAD), 6 psi. The LC–MS/MS system was operated using Analyst Instrumental Control and Data Processing software (version 1.7.2, AB Sciex) and MultiQuant software (version 3.0.3, AB Sciex).

**TABLE 1:** Mass spectrometry settings for the quantification of ketamine, norketamine, dehydronorketamine (DHNK), (2R,6R)‐hydroxynorketamine (HNK), and their deuterated internal standards.

Analyte	Type	Q1	Q3	RT	DP	EP	ce	CXP
		[Da]	[Da]	[min]	[V]	[V]	[V]	[V]
Ketamine	Quantifier	238.1	125.1	2.92	101	10	17	14
	Qualifier	238.1	89.0	2.92	101	10	45	22
Ketamine‐d4	Internal standard	242.1	129.1	2.91	46	10	33	12
Norketamine	Quantifier	224.1	125.2	2.61	46	10	21	12
	Qualifier	224.1	207.0	2.61	46	10	12	22
Norketamine‐d4	Internal standard	228.2	210.8	2.60	86	10	15	20
DHNK	Quantifier	222.1	142.2	2.51	61	10	33	12
	Qualifier	222.1	177.1	2.51	61	10	23	18
(2R,6R)‐HNK	Quantifier	240.1	124.9	1.91	61	10	29	16
	Qualifier	240.1	115.0	1.91	61	10	69	12

### 2.5 Sample Extraction

Samples were extracted by simple protein precipitation. A volume of 50 μL plasma was aliquoted and extracted with 150 μL internal standard solution (10 ng/mL ketamine‐d_4_ and 20 ng/mL norketamine‐d_4_ in acetonitrile) in Matrix tubes (0.7 mL, 96‐deepwell rack, Thermo Fisher Scientific). Afterward, the samples were vortexed for 5 min on a multitube vortexer (VX‐2500, VWR, Radnor, PA, USA). To obtain a supernatant free of precipitated plasma proteins, the samples were centrifuged for 30 min at 3220 × *g* and 10 °C.

### 2.6 Method Validation

The method was validated in accordance with the US Food and Drug Administration's (FDA) Bioanalytical Method Validation Guidance for Industry (United States Food and Drug Administration 2018), following an approach used in earlier validations (Luethi et al. 2022; Luethi et al. 2024; Thomann et al. 2022; Thomann et al. 2025; Thomann et al. 2024). The validated parameters included linearity, carryover, accuracy, precision, selectivity, sensitivity, matrix effects, extraction recovery, and analyte stability.

#### 2.6.1 Linearity

At least two CAL lines were included in each analytical run to ensure linearity. A CAL line consisted of 10 individual CAL samples ranging from 1 to 1,000 ng/mL for ketamine and norketamine, 9 CAL samples ranging from 0.25 to 100 ng/mL for DHNK, or 9 CAL samples ranging from 2.5 to 1,000 ng/mL for (2R,6R)‐HNK. A blank sample (matrix extracted with internal standard) and a double blank sample (matrix extracted with pure acetonitrile) were included prior to each CAL line. Using MultiQuant, a linear regression was generated by plotting the analyte peak area against the internal standard peak area with a weighting factor of 1/x^2^. The measured CAL concentrations were compared with the nominal CAL values, and the difference between the two values could not exceed ±15% (±20% for the LLOQ). A valid CAL line required at least 75% of the CAL points to be included, along with one LLOQ and one upper limit of quantification (ULOQ) sample; the correlation coefficient (*R*) had to be ≥ 0.99.

#### 2.6.2 Intra‐ and Inter‐Assay Accuracy and Precision

To assess intra‐ and inter‐assay accuracy and precision, seven replicates of LLOQ, LQC, MQC, and HQC samples were analyzed across three validation runs conducted on three separate days. The accuracy was assessed by calculating the percentage deviation of QC sample concentrations from their nominal values. Acceptable accuracy ranged from 85% to 115%, except for the LLOQ, where a range of 80%–120% was permitted. Precision was evaluated by calculating the coefficient of variation (%CV = [standard deviation/mean] × 100), which was required to be within ±15% (±20% for the LLOQ).

#### 2.6.3 Selectivity and Sensitivity

Method selectivity and sensitivity were evaluated using blank, double blank, and LLOQ plasma samples obtained from seven individual donors. Selectivity was considered adequate if the analyte signal at the LLOQ was at least fivefold higher than any detected noise signal in the blank and double blank samples. Sensitivity was confirmed when the mean accuracy of the LLOQ samples fell within the range of 80%–120%.

#### 2.6.4 Matrix Effect

The matrix effect was assessed by comparing the analyte peak area in plasma samples to the analyte peak area in matrix‐free samples. Plasma from seven different donors was spiked with the analytes at three QC levels (LQC, MQC, and HQC), while matrix‐free samples were prepared by spiking water with the analytes. Only a consistent matrix effect across all plasma batches and QC levels was deemed acceptable, as evidenced by a %CV of ≤ 15%.

#### 2.6.5 Extraction Recovery

Extraction recovery was evaluated by spiking blank plasma samples from seven different donors with the analyte both before and after the extraction process at the LLOQ, LQC, MQC, and HQC levels. At each QC level, the analyte peak areas obtained before and after extraction were compared. The peak area of postextraction spiked supernatants was considered to represent 100% recovery. The method was considered valid when recovery was consistent, precise, and reproducible across all QC levels and plasma batches.

#### 2.6.6 Analyte Stability

The stability of the analytes was evaluated by assessing seven replicates of LQC, MQC, and HQC samples under different storage and handling conditions. Samples were kept at room temperature for 8 h (benchtop stability), underwent 3 freeze–thaw cycles prior to analysis (freeze–thaw stability), or were stored at −20 °C or −80 °C for 3 months (medium‐term stability I and II). Two freshly prepared CAL lines and four freshly prepared sets of LQC, MQC, and HQC samples were used for the assessment.

Additionally, to assess the feasibility of reinjection following an instrument failure during an analytical run, samples were extracted, initially analyzed, and then reanalyzed after storage under one of the following conditions: in the autosampler for 24 h at 10 °C (autosampler stability), at −20 °C for 7 days (extract stability I), or at −80 °C for 7 days (extract stability II). The same acceptance criteria applied to accuracy and precision assessments were used for stability evaluation.

### 2.7 Method Application

A subset of clinical phase I study samples was analyzed to demonstrate the suitability of the validated method. The study (NCT06180759) was performed at the University Hospital Basel; it received approval from the Ethics Committee of Northwest and Central Switzerland (BASEC 2023‐01813) and was conducted in accordance with the Declaration of Helsinki and the International Conference on Harmonisation Good Clinical Practice guidelines. Six participants received an intravenous infusion of ketamine at a concentration of 1.0 mg/min for 55 min. A total of 17 blood samples were collected at predetermined time points: 0, 5, 10, 15, 20, 30, 40, 50, 55, 60, 65, 70, 75, 85, 95, 105, and 115 min. All samples were collected into lithium‐heparin‐coated S‐Monovette tubes (Sarstedt) and subsequently subjected to a centrifugal process at 1811 × *g* for a duration of 10 min to yield plasma. Thereafter, the samples were stored at −80 °C until further analysis. Pharmacokinetic parameters were calculated using Phoenix WinNonlin software (version 8.1.0, Certara, Princeton, USA).

## 3 Results and Discussion

### 3.1 Method Development

Several LC–MS methods have been developed for the analysis of ketamine and its metabolites across various biological matrices. However, many of these approaches involve lengthy analysis times or labor‐intensive extraction and sample preparation procedures (Harun et al. 2010; Hasan et al. 2017; Kurzweil et al. 2020; Moaddel et al. 2010; Rhee et al. 2021; Rochani et al. 2020; Rodriguez Rosas et al. 2003). Furthermore, not all methods include metabolites such as DHNK or (2R,6R)‐HNK, and in some cases, ketamine is analyzed as just one among dozens of compounds in broad‐spectrum screening assays (Harun et al. 2010; Kurzweil et al. 2020; Rhee et al. 2021; Rochani et al. 2020; Rodriguez Rosas et al. 2003).

In this study, we aimed to develop and optimize an LC–MS/MS method for ketamine, specifically designed for the analysis of a large number of clinical study samples. Our focus was on achieving a rapid and streamlined protocol while ensuring the inclusion of relevant ketamine metabolites.

Plasma samples were prepared and extracted using a simple protein precipitation method. A sample volume of only 50 μL was mixed with 150 μL of an internal standard solution, followed by centrifugation to obtain the supernatant, which was then injected into the HPLC system. All analytes were ionized using positive ESI mode. To optimize ionization parameters, each compound was initially introduced directly into the mass spectrometer. The two mass transitions with the highest signal intensities were selected for each analyte and designated as the quantifier and qualifier transitions. The transitions chosen for this method were largely consistent with those reported in previous studies (Harun et al. 2010; Hasan et al. 2017; Kurzweil et al. 2020; Moaddel et al. 2010; Rhee et al. 2021; Shahane et al. 2023; Toki et al. 2023). Optimal chromatographic separation of the analytes was achieved using a Symmetry C18 column (3.5 μm, 100 Å, 75 × 4.6 mm; Waters). Both (2R,6R)‐HNK and (2S,6S)‐HNK were initially tuned into the MS without significant differences in their MRM configurations. However, since the analytes could not be separated using this achiral approach, the method validation was conducted for (2R,6R)‐HNK only. A chromatogram of the separated analytes and their internal standards is shown in Figure 1.

### 3.2 Method Validation

#### 3.2.1 Method Linearity

The method demonstrated linearity across a concentration range of 1–1,000 ng/mL for ketamine and its main metabolite norketamine. For DHNK and (2R,6R)‐HNK, linear ranges of 0.25–100 and 2.5–1,000 ng/mL, respectively, were achieved. In all cases, the correlation coefficient (*R*) exceeded 0.998, indicating excellent linearity. All measured values for each analyte remained within the acceptable deviation limits from the nominal concentrations (±15% and ±20% for the LLOQ) across three analytical runs (Table S1).

#### 3.2.2 Analyte Carry‐Over

For ketamine and norketamine, a mean carry‐over of 25.7% and 25.4%, respectively, of the LLOQ peak area was observed in double blank samples following the injection of a ULOQ sample. However, in a subsequent second double blank sample, the carry‐over was reduced to 8.5% and 8.0%, respectively. For DHNK and (2R,6R)‐HNK, the carry‐over in the first double blank sample was 9.5% and 8.9%, respectively, and decreased to 3.2% and 2.9%, respectively, in the next sample. Therefore, to mitigate carry‐over for ketamine and norketamine, at least two double blank or solvent samples should be included after analyzing highly concentrated samples. In contrast, carry‐over for DHNK and (2R,6R)‐HNK remained within acceptable limits and did not necessitate additional blanks.

#### 3.2.3 Accuracy and Precision

Intra‐ and inter‐assay accuracy and precision for ketamine, norketamine, DHNK, and (2R,6R)‐HNK are presented in Table S2. For this assessment, seven replicates at each QC level (LLOQ, LQC, MQC, and HQC) were analyzed across three independent assay runs. Ketamine demonstrated inter‐assay accuracy ranging from 101.0% to 108.7%, with precision (%CV) between 2.4% and 10.2%; for norketamine, accuracy ranged from 99.5% to 118.3%, with precision between 3.2% and 10.3%; DHNK showed an accuracy of 89.8% to 109.3% and precision of 3.1% to 15.8%; (2R,6R)‐HNK exhibited accuracy between 95.0% and 106.1% and precision between 2.9% and 16.5%. Overall, inter‐assay accuracy across all analytes ranged from 93.2% to 111.9% and inter‐assay precision ranged from 3.8% to 12.6%.

#### 3.2.4 Selectivity and Sensitivity

**FIGURE 2:**
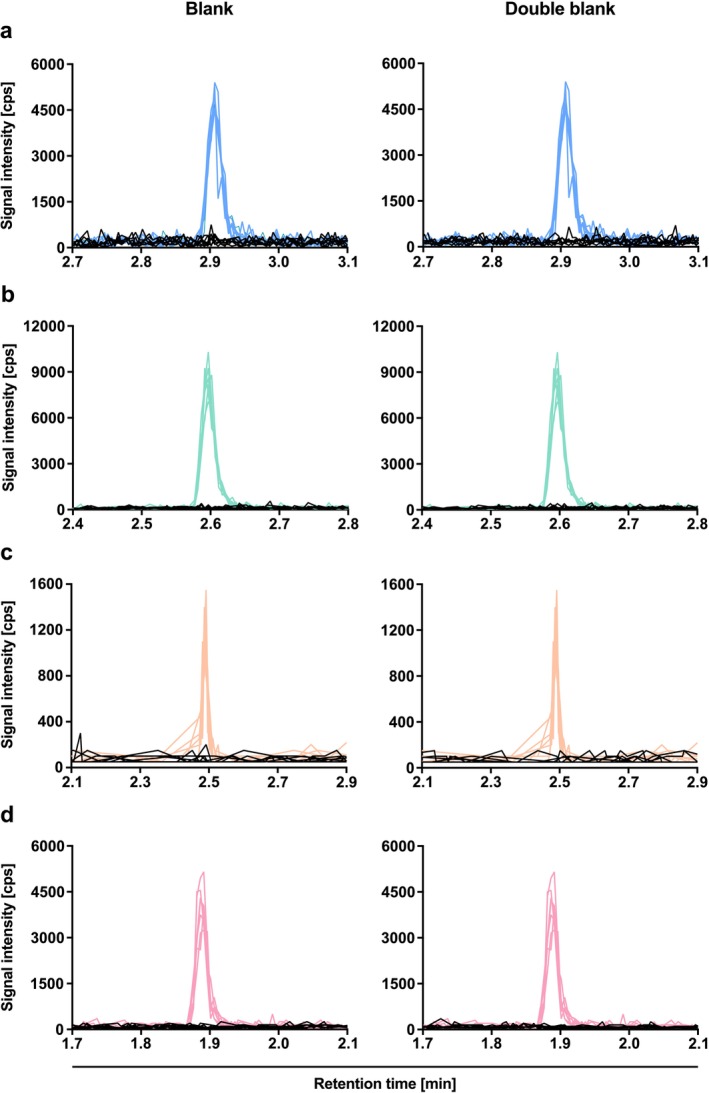
Selectivity of ketamine (a), norketamine (b), dehydronorketamine (DHNK; c), and (2R,6R)‐hydroxynorketamine (HNK; d) in human plasma. The chromatograms are displayed as an overlay of seven blank samples (left side, black lines), seven double blank samples (right side, black lines), and seven lower limit of quantification (LLOQ; colored lines) samples.

To assess selectivity and sensitivity, seven blank and double blank samples from individual donors were compared to LLOQ samples. No interference from endogenous plasma components was observed for any of the analytes, demonstrating excellent selectivity (Figure 2 and Table S3). The signal‐to‐noise ratio, determined by comparing peak areas of double blank or blank samples to those of the LLOQ, consistently exceeded 5:1 (Figure 2). Mean accuracy at the LLOQ ranged from 80.4% to 116.0%, with precision (%CV) between 6.5% and 9.3%, confirming the method's sensitivity (Table S4).

#### 3.2.5 Matrix Effect and Extraction Recovery

Matrix effect and extraction recovery results are presented in Table S5. The matrix effect was consistent for all analytes across all QC levels and plasma batches. However, a slight but consistent ion suppression was observed for DHNK and (2R,6R)‐HNK. The mean matrix effect was 102.4% for ketamine, 103.8% for norketamine, 83.9% for DHNK, and 80.7% for (2R,6R)‐HNK.

Extraction recovery was nearly complete for ketamine, norketamine, and (2R,6R)‐HNK, with mean values of 97.7%, 97.2%, and 98.2%, respectively. In contrast, DHNK showed a moderately lower recovery of 75.2%.

#### 3.2.6 Stability

The stability of ketamine, norketamine, DHNK, and (2R,6R)‐HNK under various conditions is presented in Table S6. The assessed conditions include autosampler stability, extract stability, bench‐top stability, freeze–thaw stability, and medium‐term storage stability. Across all conditions, QC levels, and analytes, accuracy ranged from 88.7% to 113.0%. The analytes were not affected by stress conditions such as 3 freeze–thaw cycles (accuracy of 90.7%–105.7%) or 8‐h bench‐top exposure (accuracy of 88.7%–104.6%), nor by storage for 3 months at either −20 °C or −80 °C (accuracy of 89.4%–113.0%). Furthermore, reinjection of extracted samples, which may be needed after an instrument failure, was feasible both the next day (accuracy of 95.7%–112.4%) and even 1 week later (accuracy of 93.4%–110.7%).

The stability of ketamine, norketamine, DHNK, and (2R,6R)‐HNK has previously been assessed by others under various storage conditions and in different matrices. In general, the stability of all compounds was maintained in biological matrices for periods ranging from several weeks to several months when stored at −20 °C or lower (Hijazi et al. 2001; Raja et al. 2024). Ketamine, formulated as a medical product for anesthesia, is deemed to be stable for several weeks even when exposed to temperatures up to 40 °C (Tran et al. 2018; Usman et al. 2024). Additionally, norketamine has demonstrated stability in whole blood at ambient temperature for a minimum of 2 h (Tran et al. 2018).

#### 3.2.7 Clinical Application

**FIGURE 3:**
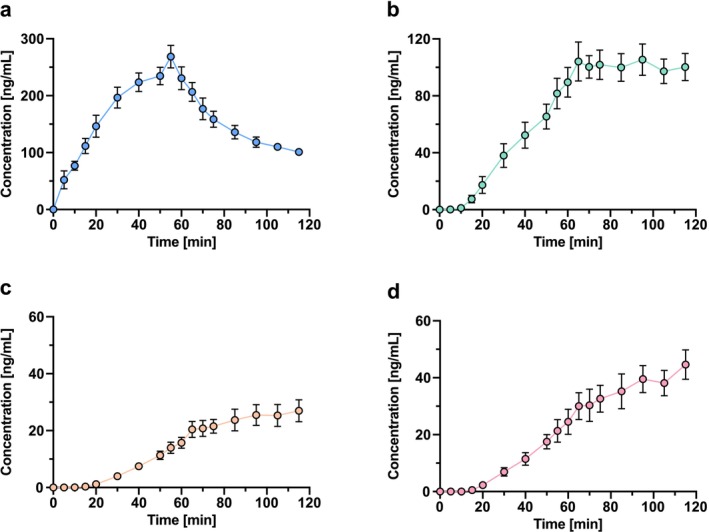
Pharmacokinetic profiles of ketamine (a), norketamine (b), dehydronorketamine (DHNK; c), and hydroxynorketamine (HNK; d) in plasma of clinical study participants (*n* = 6, mean ± SEM) receiving an intravenous infusion of 1.0 mg/min ketamine for 55 min.

To demonstrate the suitability of the developed and validated method, plasma samples of six study participants were analyzed, and the pharmacokinetic parameters were assessed. These samples were obtained from a phase I clinical trial investigating acute analgesic effects on experimentally induced nociceptive pain, hyperalgesia, and allodynia, using ketamine as a positive control. The resulting pharmacokinetic profiles of ketamine, norketamine, DHNK, and (2R,6R)‐HNK are illustrated in Figure 3 and shown in Table S7.

The maximum plasma concentration (C_max_) of ketamine was 273.8 ng/mL, reached after 53.3 min (t_max_), which coincided approximately with the end of the infusion. The plasma concentrations obtained in our study are comparable with those reported in a previous study that used a similar dosing regimen (0.5‐mg/kg body weight ketamine infusion over 40 min) (Zarate et al. 2012). In that study, ketamine plasma concentrations ranged from 177 to 204 ng/mL after 40 min, while the mean concentration in our study was 224 ng/mL at the 40‐min mark.

After the stop of the continuous infusion, two distinct plasma half‐lives (t_1/2_) were observed in our study: a short early half‐life (t_1/2⍺_) of 26 min, and a longer late half‐life (t_1/2β_) of 91 min. These values are consistent with an early distribution phase and a late elimination phase. While ketamine is typically described by a three‐compartment model, the observed kinetics are consistent with a limited sampling window in which the initial distribution phase and a combined redistribution–elimination phase are apparent, but the terminal elimination phase is not yet distinguishable (Kamp, Jonkman, et al. 2020; Kamp, Olofsen, et al. 2020). The primary observed metabolite was norketamine, which reached a C_max_ of 114 ng/mL. DHNK and (2R,6R)‐HNK exhibited lower C_max_ values of 28 and 45 ng/mL, respectively. For these metabolites, the obtained concentration values are also comparable to the results of others (Zarate et al. 2012). Due to the long plasma elimination half‐lives of these metabolites, other pharmacokinetic parameters, such as t_1/2_, could not be calculated. Furthermore, given the method's inability to differentiate between HNK isomers, quantification of racemic HNK in human plasma was performed using the validated transition for (2R,6R)‐HNK. Nevertheless, the method was successfully applied to analyze plasma samples from a clinical trial and proved suitable for the bioanalysis of ketamine, norketamine, DHNK, and HNK.

## 4 Conclusion

The developed and validated LC–MS/MS method offers a rapid, sensitive, and robust approach for the quantification of the dissociative anesthetic ketamine and its metabolites, norketamine, DHNK, and (2R,6R)‐HNK. Key advantages of the method include minimal sample volume requirements, a straightforward extraction procedure, and a fast analysis time. Validation parameters such as linearity, accuracy, precision, sensitivity, selectivity, matrix effect, extraction recovery, and stability were all within the acceptable limits defined by regulatory guidelines. As a proof of concept, the method was successfully applied to analyze pharmacokinetic samples from a clinical trial. Overall, this refined and optimized bioanalytical method facilitates both pharmacokinetic studies, drug monitoring, and forensic investigations involving ketamine and its metabolites.

## Author Contributions

**Jan Thomann:** conceptualization, data curation, methodology, investigation, validation, formal analysis, supervision, visualization, writing – original draft, writing – review and editing. **Selina Kraus:** investigation, validation, formal analysis, visualization, writing – original draft, writing – review and editing. **Livio Erne:** investigation, resources, writing – review and editing. **Severin B. Vogt:** formal analysis, investigation, resources, writing – review and editing. **Matthias E. Liechti:** funding acquisition, writing – review and editing. **Dino Luethi:** conceptualization, data curation, methodology, investigation, validation, formal analysis, supervision, writing – review and editing.

## Conflicts of Interest

The authors declare no conflicts of interest.

## Supporting information


**Table S1.** Linearity of calibration lines in plasma containing ketamine, norketamine, dehydronorketamine (DHNK), and (2R,6R)‐hydroxynorketamine (HNK).
**Table S2.** Accuracy and precision of ketamine, norketamine, dehydronorketamine (DHNK), and (2R,6R)‐hydroxynorketamine (HNK) QC samples in plasma.
**Table S3.** Selectivity of ketamine, norketamine, dehydronorketamine (DHNK), and (2R,6R)‐hydroxynorketamine (HNK) in plasma.
**Table S4.** Sensitivity of ketamine, norketamine, dehydronorketamine (DHNK), and (2R,6R)‐hydroxynorketamine (HNK) in plasma.

## Data Availability

Data that supports the findings of this study are available in the Supporting Information of this article.
