# Supplementary material for: Development and Validation of a Rapid LC–MS/MS Method for Plasma Analysis of Ketamine, Norketamine, Dehydronorketamine, and Hydroxynorketamine
Source: Biomed Chromatogr. 2025 Jul 21;39(9):e70171. doi: 10.1002/bmc.70171 (PMC12280840; doi:10.1002/bmc.70171)
Supplement: Supplementary file 1 — Table S1. Linearity of calibration lines in plasma containing ketamine, norketamine, dehydronorketamine (DHNK), and (2R,6R)‐hydroxynorketamine (HNK). Table S2. Accuracy and precision of ketamine, norketamine, dehydronorketamine (DHNK), and (2R,6R)‐hydroxynorketamine (HNK) QC samples in plasma. Table S3. Selectivity of ketamine, norketamine, dehydronorketamine (DHNK), and (2R,6R)‐hydroxynorketamine (HNK) in plasma. Table S4. Sensitivity of ketamine, norketamine, dehydronorketamine (DHNK), and (2R,6R)‐hydroxynorketamine (HNK) in plasma. [file BMC-39-e70171-s001.pdf]

Supplementary data to:

Development and validation of a rapid LC–MS/MS method for plasma analysis of ketamine, norketamine, dehydronorketamine, and hydroxynorketamine

Jan Thomann[1,2,+], Selina Kraus[1,2,+], Livio Erne[2,3], Severin B. Vogt[2,3], Matthias E. Liechti[1,2,3], Dino Luethi[1,2,*]

[1]Division of Clinical Pharmacology and Toxicology, Department of Biomedicine, University Hospital Basel, Switzerland
[2]Division of Clinical Pharmacology and Toxicology, Department of Pharmaceutical Sciences, University of Basel, Switzerland
[3]Division of Clinical Pharmacology and Toxicology, Department of Clinical Research, University Hospital Basel, Switzerland
[+]These authors contributed equally to this work

*Corresponding author: Dr. Dino Luethi, Division of Clinical Pharmacology and Toxicology, University Hospital Basel, Hebelstrasse 20, 4031 Basel, Switzerland. E-mail: dino.luethi@unibas.ch

Supplementary Table 1. Linearity of calibration lines in plasma containing ketamine, norketamine, dehydronorketamine (DHNK), and (2R,6R)-hydroxynorketamine (HNK).

Analyte	Nominal concentration	Assay 1		Assay 2		Assay 3	
	[ng/mL]	Cø [ng/mL]	%Accuracy	Cø [ng/mL]	%Accuracy	Cø [ng/mL]	%Accuracy
Ketamine	1	1.0	102.6	1.0	101.5	1.0	103.2
	2.5	2.4	94.9	2.5	100.0	2.4	94.4
	5	4.9	97.0	4.7	94.8	4.8	96.5
	10	9.6	96.1	9.2	92.4	9.7	97.2
	25	27.9	111.7	26.3	105.3	24.6	98.3
	50	50.5	101.1	52.1	104.1	51.1	102.1
	100	101.0	101.0	101.7	101.7	105.0	105.0
	250	249.3	99.7	257.7	103.1	260.0	104.0
	500	498.1	99.6	504.1	100.8	501.9	100.4
	1,000	964.2	96.4	963.7	96.4	990.3	99.0
Norketamine	1	1.0	100.5	1.0	100.7	1.0	99.5
	2.5	2.4	96.7	2.4	97.4	2.5	99.0
	5	5.0	100.9	5.0	99.1	5.2	103.7
	10	10.3	103.0	10.3	102.7	10.1	101.1
	25	26.3	105.0	26.5	106.0	25.5	102.0
	50	54.8	109.6	52.2	104.4	50.1	100.2
	100	100.4	100.4	96.3	96.3	103.8	103.8
	250	254.6	101.8	252.1	100.9	253.9	101.6
	500	448.2	89.6	505.3	101.1	493.5	98.7
	1,000	924.6	92.5	915.7	91.6	905.7	90.6
DHNK	0.25	0.2	93.0	0.3	106.1	0.3	103.7
	0.5	0.5	103.7	0.5	96.5	0.5	90.7
	1	1.1	106.0	0.9	92.9	0.9	94.1
	2.5	2.5	99.5	2.6	103.8	2.5	100.4
	5	5.3	105.8	5.1	102.7	5.0	100.2
	10	9.8	97.8	9.8	98.0	10.2	102.3
	25	24.7	98.9	25.7	102.9	25.3	101.2
	50	48.3	96.6	50.9	101.8	50.1	100.1
	100	95.1	95.1	98.4	98.4	102.7	102.7
(2R,6R)-HNK	2.5	2.6	101.9	2.5	101.8	2.6	102.4
	5	4.7	94.0	4.9	97.0	4.9	98.0
	10	10.4	104.1	9.9	98.8	9.3	92.9
	25	24.8	99.2	24.9	99.7	24.3	97.1
	50	51.6	103.2	50.7	101.3	50.8	101.7
	100	99.4	99.4	99.7	99.7	102.4	102.4
	250	251.3	100.5	244.4	97.8	253.3	101.3
	500	474.3	94.9	524.8	105.0	505.7	101.1
	1,000	1,024.1	102.4	990.4	99.0	1,021.4	102.1

Supplementary Table 2. Accuracy and precision of ketamine, norketamine, dehydronorketamine (DHNK), and (2R,6R)-hydroxynorketamine (HNK) QC samples in plasma.

Analyte	QC level	Nominal concentration	Assay 1			Assay 2			Assay 3			Inter-assay		
		[ng/mL]	Cø [ng/mL]	%Accuracy	%CV	Cø [ng/mL]	%Accuracy	%CV	Cø [ng/mL]	%Accuracy	%CV	Cø [ng/mL]	%Accuracy	%CV
Ketamine	LLOQ	1	1.0	101.0	8.8	1.1	107.1	10.2	1.1	111.0	9.7	1.1	106.4	10.0
	LQC	2.5	2.6	104.8	7.0	2.6	104.0	4.6	2.6	102.8	10.1	2.6	103.9	7.2
	MQC	50	53.3	106.7	4.1	52.3	104.6	2.4	54.3	108.7	4.0	53.3	106.7	3.8
	HQC	500	524.4	104.9	5.5	522.0	104.4	4.4	542.8	108.6	2.5	529.7	106.0	4.5
Norketamine	LLOQ	1	1.0	103.7	7.9	1.2	118.3	10.3	1.1	113.6	5.5	1.1	111.9	9.7
	LQC	2.5	2.7	107.2	8.0	2.8	110.8	4.1	2.7	107.5	7.5	2.7	108.5	6.5
	MQC	50	53.0	106.0	6.4	56.4	112.7	3.2	56.0	112.1	5.6	55.1	110.3	5.7
	HQC	500	497.6	99.5	4.0	525.4	105.1	5.2	508.1	101.6	3.2	510.4	102.1	4.6
DHNK	LLOQ	0.25	0.3	109.3	9.6	0.3	109.2	15.8	0.3	100.5	7.2	0.3	106.3	12.0
	LQC	0.5	0.5	97.0	12.3	0.5	100.3	9.2	0.5	91.9	13.1	0.5	96.7	11.5
	MQC	5	4.7	93.4	7.3	4.5	89.8	9.0	4.9	98.7	3.1	4.7	93.9	7.6
	HQC	50	45.7	91.5	7.0	47.8	95.5	4.4	46.3	92.6	5.8	46.6	93.2	5.8
(2R,6R)-HNK	LLOQ	2.5	2.6	102.5	10.2	2.7	106.1	16.5	2.4	97.4	9.8	2.6	102.0	12.6
	LQC	5	5.0	100.8	6.4	5.1	101.1	9.0	4.8	95.0	6.5	5.0	98.9	7.6
	MQC	50	49.3	98.6	4.2	51.1	102.1	5.8	52.3	104.5	5.8	50.9	101.8	5.6
	HQC	500	493.8	98.8	10.0	505.4	101.1	4.3	522.4	104.5	2.9	507.2	101.4	6.5

HQC, high quality control; LLOQ, lower limit of quantification; LQC, low quality control; MQC, mid quality control.

Supplementary Table 3. Selectivity of ketamine, norketamine, dehydronorketamine (DHNK), and (2R,6R)-hydroxynorketamine (HNK) in plasma.

Analyte	Condition	Subject 1		Subject 2		Subject 3		Subject 4		Subject 5		Subject 6		Subject 7	
		Peak area	%LLOQ	Peak area	%LLOQ	Peak area	%LLOQ	Peak area	%LLOQ	Peak area	%LLOQ	Peak area	%LLOQ	Peak area	%LLOQ
Ketamine	LLOQ	6,022	-	5,580	-	6,696	-	5,628	-	5,999	-	5,056	-	5,227	-
	Double Blank	0	0.0	123	2.2	0	0.0	0	0.0	449	7.5	0	0.0	0	0.0
	Blank	82	1.4	0	0.0	0	0.0	0	0.0	0	0.0	0	0.0	0	0.0
Norketamine	LLOQ	16,840	-	16,570	-	17,890	-	14,510	-	16,840	-	17,230	-	17,640	-
	Double Blank	691	4.1	0	0.0	315	1.8	10	0.1	0	0.0	0	0.0	0	0.0
	Blank	0	0.0	36	0.2	22	0.1	0	0.0	109	0.6	0	0.0	0	0.0
DHNK	LLOQ	2,007	-	2,704	-	2,374	-	2,346	-	2,772	-	2,533	-	2,770	-
	Double Blank	0	0.0	0	0.0	0	0.0	0	0.0	0	0.0	0	0.0	0	0.0
	Blank	0	0.0	0	0.0	0	0.0	29	1.2	0	0.0	0	0.0	0	0.0
(2R,6R)-HNK	LLOQ	4,389	-	5,543	-	4,596	-	4,929	-	4,382	-	4,792	-	5,034	-
	Double Blank	0	0.0	0	0.0	137	3.0	0	0.0	118	2.7	0	0.0	56	1.1
	Blank	23	0.5	0	0.0	0	0.0	40	0.8	13	0.3	7.0	0.1	12	0.2

LLOQ, lower limit of quantification.

Supplementary Table 4. Sensitivity of ketamine, norketamine, dehydronorketamine (DHNK), and (2R,6R)-hydroxynorketamine (HNK) in plasma.

Analyte		Nominal concentration [ng/mL]	Cø [ng/mL]	%Accuracy	%CV
Ketamine	Plasma 1	1.00	0.97	97.0	
	Plasma 2	1.00	0.91	91.0	
	Plasma 3	1.00	1.07	107.0	
	Plasma 4	1.00	0.96	96.0	
	Plasma 5	1.00	0.99	99.0	
	Plasma 6	1.00	0.84	84.0	
	Plasma 7	1.00	0.86	86.0	
	Mean	1.00	0.94	94.3	8.7
Norketamine	Plasma 1	1.00	1.02	102.0	
	Plasma 2	1.00	1.06	106.0	
	Plasma 3	1.00	1.11	111.0	
	Plasma 4	1.00	0.91	91.0	
	Plasma 5	1.00	1.09	109.0	
	Plasma 6	1.00	1.10	110.0	
	Plasma 7	1.00	1.09	109.0	
	Mean	1.00	1.05	105.4	6.5
DHNK	Plasma 1	0.25	0.22	88.0	
	Plasma 2	0.25	0.28	112.0	
	Plasma 3	0.25	0.26	104.0	
	Plasma 4	0.25	0.24	96.0	
	Plasma 5	0.25	0.29	116.0	
	Plasma 6	0.25	0.26	104.0	
	Plasma 7	0.25	0.28	112.0	
	Mean	0.25	0.26	104.7	9.3
(2R,6R)-HNK	Plasma 1	2.50	2.01	80.4	
	Plasma 2	2.50	2.51	100.4	
	Plasma 3	2.50	2.18	87.2	
	Plasma 4	2.50	2.21	88.4	
	Plasma 5	2.50	2.02	80.8	
	Plasma 6	2.50	2.11	84.4	
	Plasma 7	2.50	2.25	90.0	
	Mean	2.50	2.19	87.4	7.9

Supplementary Table 5. Matrix effect and extraction recovery for ketamine, norketamine, dehydronorketamine (DHNK), and (2R,6R)-hydroxynorketamine (HNK) in plasma.

Analyte	QC level	Matrix effect	Recovery
		Mean [%]	Mean [%]
Ketamine	LQC	103.9	101.0
	MQC	99.8	100.4
	HQC	103.5	91.7
	Mean	102.4	97.7
Norketamine	LQC	106.6	100.8
	MQC	100.1	100.6
	HQC	104.8	90.3
	Mean	103.8	97.2
DHNK	LQC	82.3	77.8
	MQC	78.4	76.7
	HQC	91.1	71.1
	Mean	83.9	75.2
(2R,6R)-HNK	LQC	81.0	107.2
	MQC	73.6	100.2
	HQC	87.4	87.1
	Mean	80.7	98.2

HQC, high quality control; LQC, low quality control; MQC, mid quality control.

Supplementary Table 6. Stability of ketamine, norketamine, dehydronorketamine (DHNK), and (2R,6R)-hydroxynorketamine (HNK) in plasma under different conditions.

Condition	QC level	Ketamine	Norketamine	DHNK	(2R,6R)-HNK
		%Accuracy	%Accuracy	%Accuracy	%Accuracy
Autosampler stability (1 day at 10 °C)	LQC	108.3	105.5	98.3	112.4
	MQC	105.1	107.8	106.0	101.1
	HQC	102.2	99.3	100.5	95.7
Extract stability I (7 days at -20 °C)	LQC	106.3	109.9	108.6	93.4
	MQC	110.7	109.0	97.6	96.8
	HQC	106.5	101.5	107.4	104.3
Extract stability II (7 days at -80 °C)	LQC	108.6	112.2	96.9	94.6
	MQC	109.0	110.3	94.5	94.8
	HQC	109.4	101.2	100.3	102.0
Benchtop stability (8 h at RT)	LQC	101.3	103.2	98.9	88.7
	MQC	100.4	104.3	98.5	93.1
	HQC	104.6	97.0	102.7	94.1
Freeze-thaw stability (3 freeze-thaw cycles)	LQC	105.7	102.9	94.0	90.7
	MQC	102.7	105.2	94.4	93.0
	HQC	100.0	101.2	95.0	98.2
Medium-term stability I (3 months at -20 °C)	LQC	98.4	102.1	89.4	111.4
	MQC	103.8	108.3	91.8	106.3
	HQC	102.3	100.2	96.3	105.9
Medium-term stability II (3 months at -80 °C)	LQC	97.9	108.6	106.3	102.4
	MQC	102.2	102.1	104.8	100.2
	HQC	102.5	99.9	113.0	99.7

HQC, high quality control; LQC, low quality control; MQC, mid quality control; RT, room temperature

Supplementary Table 7. Pharmacokinetic parameters in clinical study samples from six participants following a 55-min infusion of ketamine at 1 mg/min.

Analyte	C_{max} (range) [ng/mL]	t_{max} (range) [min]	$t_{1/2\alpha}$ (range) [min]	$t_{1/2\beta}$ (range) [min]
Ketamine	273.8 (225.1–360.9)	53.3 (50.0–55.0)	25.6 (14.7–49.9)	90.8 (43.4–136.3)
Norketamine	113.7 (84.6–161.5)	87.5 (65.0–115.0)	-	-
DHNK	27.7 (16.1–43.4)	105.0 (85.0–115.0)	-	-
HNK	44.6 (29.4–62.9)	115.0 (115.0–115.0)	-	-

C_{max}, maximum concentration; t_{max}, time to reach maximum concentration; $t_{1/2\alpha}$, early half-life; $t_{1/2\beta}$, late half-life.
